# *Schistocephalus* parasite infection alters sticklebacks’ movement ability and thereby shapes social interactions

**DOI:** 10.1038/s41598-020-69057-0

**Published:** 2020-07-23

**Authors:** Jolle W. Jolles, Geoffrey P. F. Mazué, Jacob Davidson, Jasminca Behrmann-Godel, Iain D. Couzin

**Affiliations:** 1 Department of Collective Behaviour, Max Planck Institute of Animal Behavior, Konstanz, Germany; 2 Zukunftskolleg, University of Konstanz, Konstanz, Germany; 3 Centre for the Advanced Study of Collective Behaviour, University of Konstanz, Konstanz, Germany; 4 School of Life and Environmental Sciences, University of Sydney, Sydney, Australia; 5 Limnological Institute, University of Konstanz, Konstanz, Germany

**Keywords:** Behavioural ecology, Animal behaviour

## Abstract

Parasitism is ubiquitous in the animal kingdom. Although many fundamental aspects of host-parasite relationships have been unravelled, few studies have systematically investigated how parasites affect organismal movement. Here we combine behavioural experiments of *Schistocephalus solidus* infected sticklebacks with individual-based simulations to understand how parasitism affects individual movement ability and thereby shapes social interaction patterns. High-resolution tracking revealed that infected fish swam, accelerated, and turned more slowly than did non-infected fish, and tended to be more predictable in their movements. Importantly, the strength of these effects increased with increasing parasite load (proportion of body weight), with more heavily infected fish showing larger changes and impairments in behaviour. When grouped, pairs of infected fish moved more slowly, were less cohesive, less aligned, and less temporally coordinated than non-infected pairs, and mixed pairs were primarily led by the non-infected fish. These social patterns also emerged in simulations of self-organised groups composed of individuals differing similarly in speed and turning tendency, suggesting infection-induced changes in mobility and manoeuvrability may drive collective outcomes. Together, our results demonstrate how infection with a complex life-cycle parasite affects the movement ability of individuals and how this in turn shapes social interaction patterns, providing important mechanistic insights into the effects of parasites on host movement dynamics.

## Introduction

Parasitism is ubiquitous across the animal kingdom, with parasites often exerting considerable influence on their hosts by consuming energy and inducing morphological, physiological, and behavioural changes^1–3^. Parasites have been best studied in terms of their ecological and evolutionary effects on hosts^1,4^, but may also have large effects on host behaviour^2^. Besides potential manipulation of the host, parasites also often change their host’s morphology and physiology, which may strongly alter its movement dynamics^2,5^, such as by reducing the energy available to allocate to movement or by compromising their movement capacity and mobility. So far, few studies have systematically investigated the mechanistic basis of such potential behavioural modifications^2,5^ or considered the repercussions this may have for social interactions and collective outcomes via self-organising effects^6,7^.

**Figure 1:**
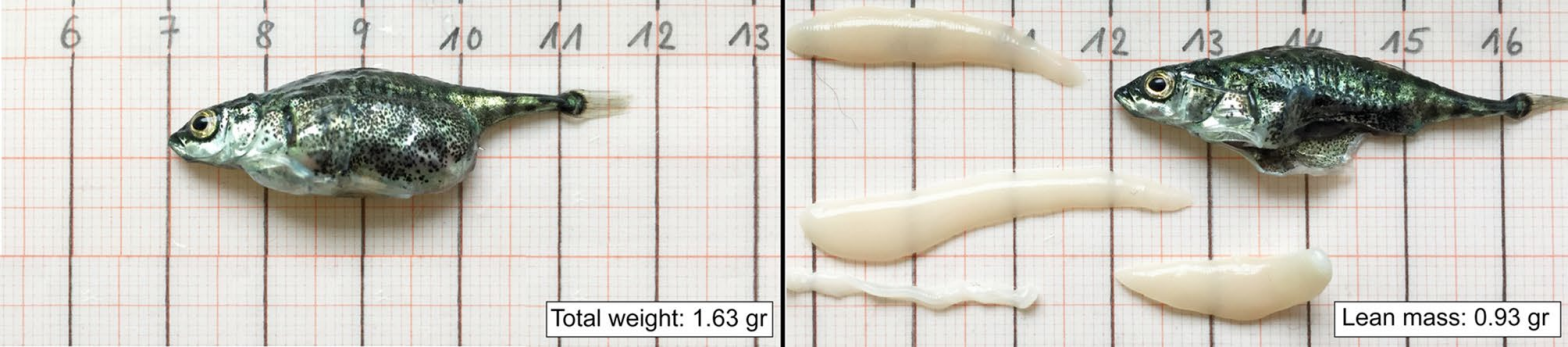
A heavily infected stickleback*.* A euthanatized, heavily infected stickleback before (left) and after (right) the removal of the four *S. solidus* plerocercoids it carried. Photos by J. W. Jolles.

Three-spined sticklebacks (*Gasterosteus aculeatus*) infected with the flatworm *Schistocephalus solidus* are a host-parasite model system with well-documented parasite effects on host behaviour^8,9^. *S. solidus* is a complex life-cycle parasite that has to sequentially infect a copepod, the three-spined stickleback, and a fish-eating bird to survive and reproduce. It is often proposed that parasites with complex life cycles manipulate their host’s behaviour in order to increase their probability of transmission to their final host^4^. However, the parasite may also affect behaviour via energetic and physical constraints. The plerocercoids of *S. solidus* grow very rapidly in the body cavity of their host and thereby incur significant energetic demands: infected individuals have a poorer body condition and lower energetic reserves^10,11^, a higher metabolic rate^12–14^, and lower growth rates (in terms of lean mass)^10,11^ compared to non-infected fish. The plerocercoids can grow so large that they may weigh as much as their host^15^ and grossly distend their body^11,16,17^ (see Fig. 1), which may result in strong impairments in mobility and manoeuvrability.

Considerable work exists on the effects of *S. solidus* on the behaviour of infected sticklebacks. The parasite strongly shifts how sticklebacks trade-off anti-predator and foraging behaviour, with infected fish exhibiting reduced predator avoidance and an increased motivation to feed^18–27^, and these effects get stronger with increasing parasite load^18,20,26^. In terms of mobility, researchers have long noted that infected sticklebacks move more slowly than non-infected fish^10,13,15,18^, but the evidence remains mostly anecdotal. Furthermore, work investigating the total distance moved by freely moving sticklebacks did not find significant differences between infected and non-infected fish^14,28^. Forced swimming tests revealed that infected fish tend to have reduced maximum swim speeds and endurance^13,29,30^, and exhibit increasingly high metabolic costs when forced to move at greater speeds^12–14^. However, it remains unclear if the parasite may impair turning mobility and how these constraints influence individual movement decisions.

In general there is still little mechanistic consideration of the effects that parasites, like *S. solidus,* may have on the movement dynamics and decisions of their host^31^. Such knowledge is especially important because the behavioural changes that parasites may induce at the individual level may have large repercussions at the group and community level, with in turn wide-ranging ecological and evolutionary consequences^32^. Indeed, a number of studies, in fish in particular, have shown that parasites may affect fishes’ tendency to shoal within-group positioning and leadership, group cohesion and movement dynamics, and among-group assortment^6,7,33–39^. Work using the stickleback-*S. solidus* system furthermore suggests that infected fish may have weaker social responses^33,37^ that may influence the risk-taking behaviour of non-infected group mates^37^. However, so far it remains unclear exactly how such social patterns emerge from individual differences in parasite infection.

Here we systematically studied the fine-scale movements of individuals and pairs of sticklebacks with different levels of *S. solidus* infection to get a better understanding of how parasite infection alters host movement dynamics and how that in turn may influence social interaction patterns. We thereby focused on parasite load (as proportion of fish’s body weight), rather than just their infection status, to better understand the mechanistic underpinnings. We ran two experiments in which we evaluated fish’s movements when able to swim freely in an open environment, both individually (‘solo assay’) and in pairs (‘pair assay’), and when being chased and startled. We subsequently used high-resolution tracking to objectively quantify fish’s individual and pair movement characteristics. We hypothesised that infected fish would be slower and less mobile than non-infected fish and furthermore that these effects would be more pronounced the higher their parasite load because of increased energetic demands and decreased movement capacity due to the parasite. We predicted that due to self-organising effects, these individual-level effects would have key impacts on group behaviour, with pairs of infected fish predicted to be slower, less cohesive and less coordinated than non-infected pairs, and mixed pairs to be primarily led by the likely faster, non-infected fish. To further test these predictions, and to seek a parsimonious mechanistic explanation for the observed patterns, we compared our empirical data to individual-based simulations of self-organised groups with individuals differing similarly in speed, turning ability, and continuity of movement as did the fish in our experiments.

## Results

### How does *S. solidus* infection affect individual movement?

**Figure 2:**
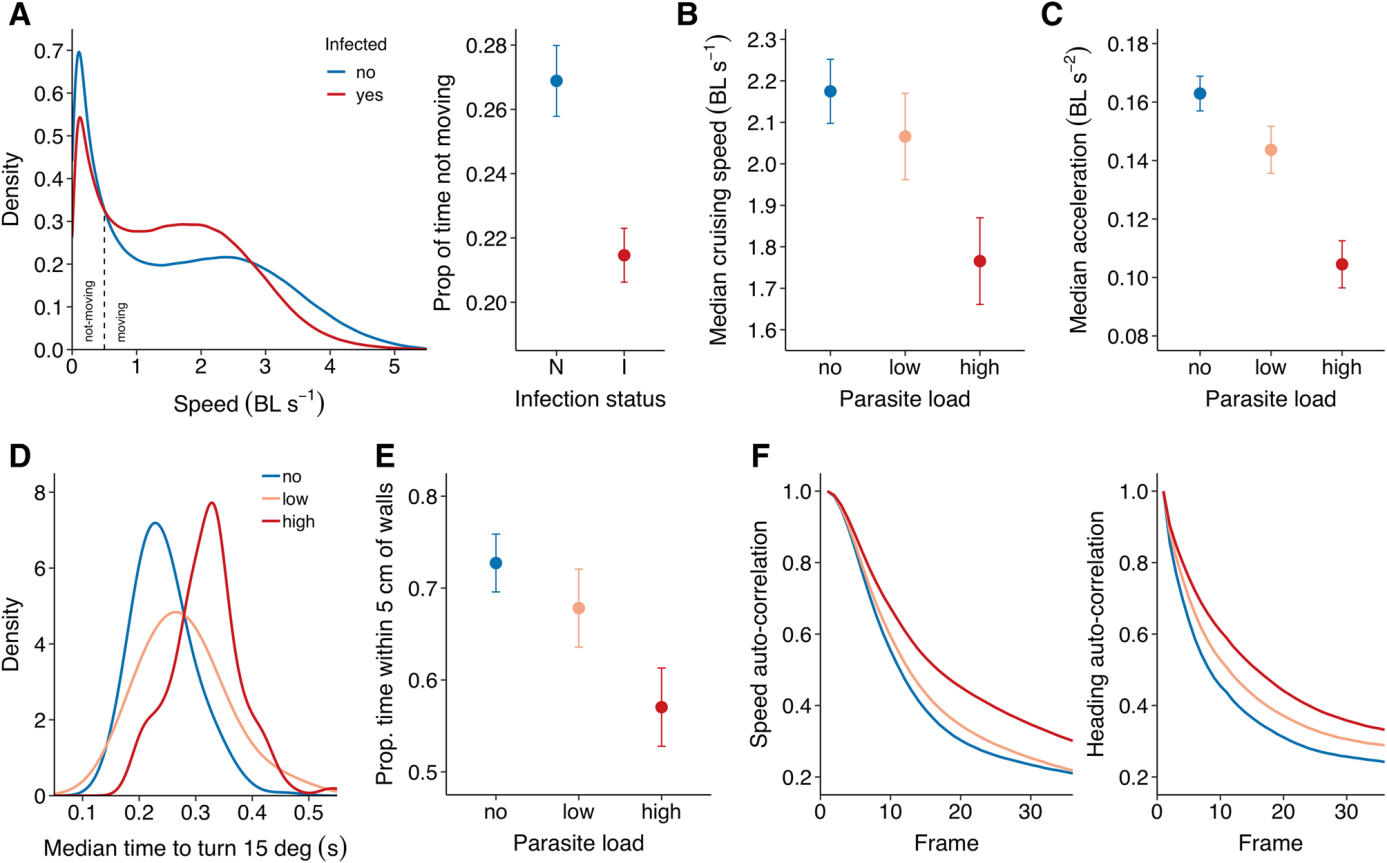
Movement characteristics of fish in the solo assay. (**A**) Density plots of the speed distribution of infected (red; *n* = 94) and non-infected (blue; *n* = 86) fish (left), based on the full frame-by-frame dataset, and the proportion of time fish were in the not-moving state (< 0.5 BL s^−1^) in terms of their infection status (right). (**B**) Fish’s cruising speed, i.e. their median speed when in the moving state, and (**C**) mean (tangential) acceleration for fish with no (*n* = 86), low (*n* = 47), and high (*n* = 47) parasite load (referred to as non-, slightly-, and heavily-infected fish respectively). (**D**) Density plots of the median time it took fish to turn their body at least 15°, (**E**) fish’s ‘thigmotaxis’, the proportion of time spent within 5 cm of the nearest wall, in terms of their parasite load, and (**F**) auto-correlation plots of fish’s speed (left) and heading (right) in terms of their parasite load. Error bars are 95% confidence intervals of the mean.

When moving freely in an open environment, infected fish were less variable in their movement speed and exhibited both lower maximum speeds and a higher tendency to keep moving compared to non-infected fish (Fig. 2A). Parasite load, categorized as no (‘non-infected’; *n* = 84), low (‘slightly infected’; 0.002–0.27, *n* = 47), and high (‘heavily infected’; 0.27—0.49; *n* = 47), had a strong effect on both the cruising speed (median speed when moving; χ^2^ = 35.68, *p* < 0.001) and mean acceleration (χ^2^ = 100.3, *p* < 0.001) of the fish: heavily infected fish swam and accelerated more slowly than did slightly- and non-infected fish (Fig. 2B,C, Supplementary Fig. S6). Fish’s body weight had no significant effect on their acceleration (χ^2^ = 1.20, *p* = 0.273).

Although on a second-by second basis parasite load did not influence the maximum turning rate of the fish (χ^2^ = 1.33, *p* = 0.514), heavily infected fish took longer to make a more abrupt change in their orientation (> 15°) than did slightly- and non-infected fish (χ^2^ = 52.60, *p* < 0.001; Fig. 2D). Such differences in mobility may also affected fish’s ability to stay close to the tank walls (thigmotaxis), a behaviour known to be positively linked with anxiety^40^, and change their movements over time. We found that non-infected fish spent the most and heavily infected fish the least amount of time within 5 cm of the tank walls (χ^2^ = 31.59, *p* < 0.001; Fig. 2E) and that they were the fastest to change in speed (χ^2^ = 53.01, *p* < 0.001) and heading (χ^2^ = 81.51, *p* < 0.001), with consequentially heavily infected fish being the most predictable in their movements (Fig. 2F). As turning behaviour is affected by speed, we also looked at how quickly fish changed their heading over distance rather than over time, revealing that the effect of predictability in turning behaviour, while being less strong, remained (χ^2^ = 13.39, *p* = 0.001).

**Figure 3:**
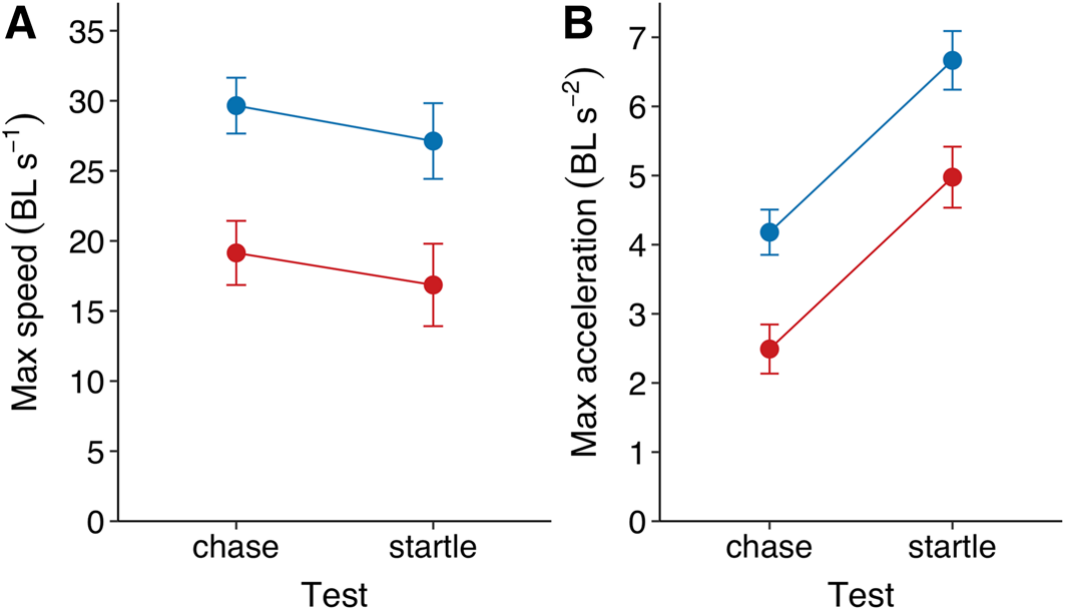
Fish’s speed and acceleration in the chase and startle assay. (**A**) Maximum speed and (**B**) maximum acceleration in terms of body length for infected (red; *n* = 11) and non-infected (blue; *n* = 13) fish. Maxima were quantified based on .75 quantile probabilities. Error bars are 95% confidence intervals of the mean.

Fish that were chased or startled exhibited on average much higher speeds and acceleration than fish in the free-movement solo assay (Figs. 2 and 3). Under these high-demand conditions, infected fish were again found to be the slowest, reaching a significantly lower maximum speed (χ^2^ = 24.33, *p* < 0.001) and lower maximum acceleration (χ^2^ = 29.14, *p* < 0.001) than non-infected fish (Fig. 3, Supplementary Figs. S3, S4). Non-infected fish reached their maximum speed much earlier than non-infected fish when being chased, and were able to swim considerably further away in the first moments after being startled (Supplementary Fig. S7).

### How does *S. solidus* infection affect social interactions?

**Figure 4:**
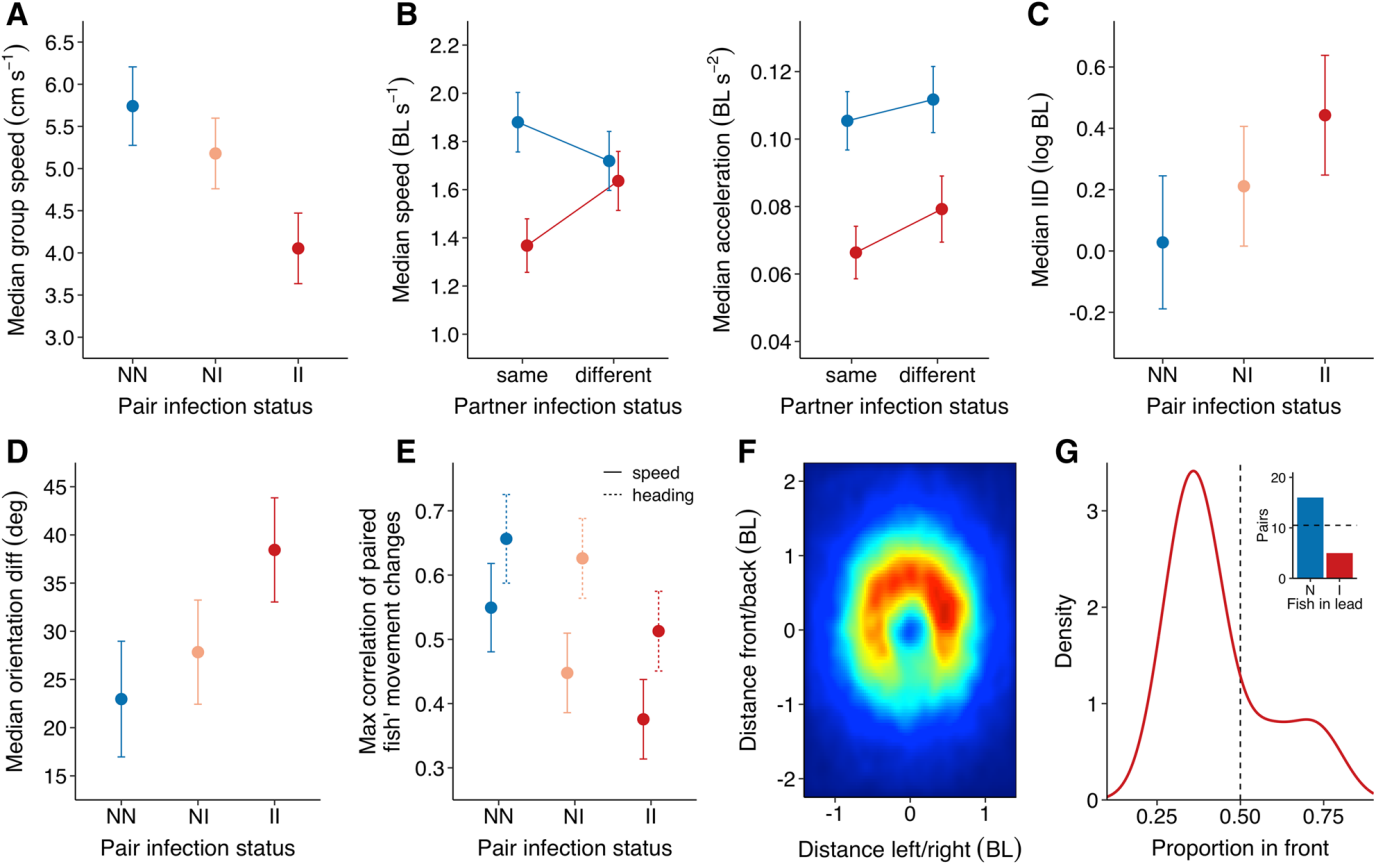
Movement characteristics and social interactions in the pair assay. (**A**) Median movement speed of the group centroid in cm/s for pairs in which neither fish was infected (NN, *n* = 17), one fish was infected (NI, *n* = 21) of both fish were infected (II; *n* = 21). (**B**) Fish’s median cruising speed in BL/s and median acceleration in BL/s^2^ in terms of fish’s infection status, and the relative infection status of their partner. (**C**) Relationship between pairs’ infection status and their median inter-individual distance in average body length (log-transformed), (**D**) their median difference in orientation, and (**E**) the max correlation coefficient of fish’s speed (solid line) and heading changes (dashed line). Error bars are 95% confidence intervals. (**F**) Heatmap of the spatial positioning of infected fish’s partner in the mixed pairs based on the frame-by-frame data. Data is cropped to 72% of the full parameter space to show the most relevant area only, with the colour scale being proportional to the densest bin of the plot (blue = low; red = high). (**G**) Density plot of the proportion of time infected fish spent in front in the mixed pairs, with inset showing the number of pairs for which the non-infected (N) or the infected (I) fish predominantly led (*n* = 21 mixed pairs).

Although the fish in each pair strongly conformed in their speed, relative inter-individual differences in speed were maintained across the solo and pair assays (*R*_C_ = 0.62, 95% CI 0.49–0.72; see Supplementary Fig. S8). Pairs’ infection status has a significant effect on their movement speed (*F*_2,56_ = 15.59, *p* < 0.001), with pairs consisting of two infected fish swimming the slowest and pairs of two non-infected fish the fastest (Fig. 4A). Despite considerable variation in size among the pairs (see Supplementary Fig. S2), this variation did not help better explain their joint movement speed (ΔAIC = 1.43). As in the solo assay, infected fish swam more slowly than non-infected fish, but only so in the homogeneous pairs (status × relative partner status: χ^2^ = 17.95, *p* < 0.001), showing that both infected and non-infected fish in the mixed pairs adjusted their swimming speed in response to their partner (Fig. 4B). Such an effect was not found for fish’s acceleration (χ^2^ = 0.63, *p* = 0.427) and instead, infected fish accelerated on average more slowly than did non-infected fish irrespective of their partner (χ^2^ = 56.92, *p* < 0.001; Fig. 4B). Importantly, our high-resolution tracking revealed that fish exhibited different social response patterns depending on their infection status (Supplementary Fig. S9), with infected fish showing smaller speed and turning changes in response to their partner.

The infection status of the pair also significantly affected their cohesion (*F*_2,56_ = 4.13, *p* = 0.021, Fig. 4C) and alignment (*F*_2,56_ = 7.97, *p* < 0.001, Fig. 4D), with pairs of non-infected fish being the most cohesive and aligned, and pairs of infected fish the least so. Furthermore, while at very low speeds, pairs of non-infected fish still stayed relatively well aligned, this was less the case for infected pairs (Supplementary Fig. S10). Pairs’ infection status furthermore had a significant effect on their temporal coordination, both in terms of how paired individuals modified their speed (*F*_2,56_ = 7.09, *p* = 0.002) and their orientation (*F*_2,56_ = 5.62, *p* = 0.006) in response to their partner, with infected pairs being the least and non-infected pairs the most coordinated (Fig. 4E). Also spatial leadership of the mixed pairs was linked to infection status, with non-infected fish being predominantly leading (Fig. 4F,G), both in terms of how often this was seen in pairs of fish (χ^2^ = 5.76, *p* = 0.016) and in the average proportion of time spent in front (*t*_20_ = 2.11, *p* = 0.024). Leadership was strongly linked to differences in speed (Supplementary Fig. S11), with infected fish swimming more slowly when in the front position compared to non-infected fish (Supplementary Fig. S10A).

### Simulations of self-organising effects of individual heterogeneity

**Figure 5:**
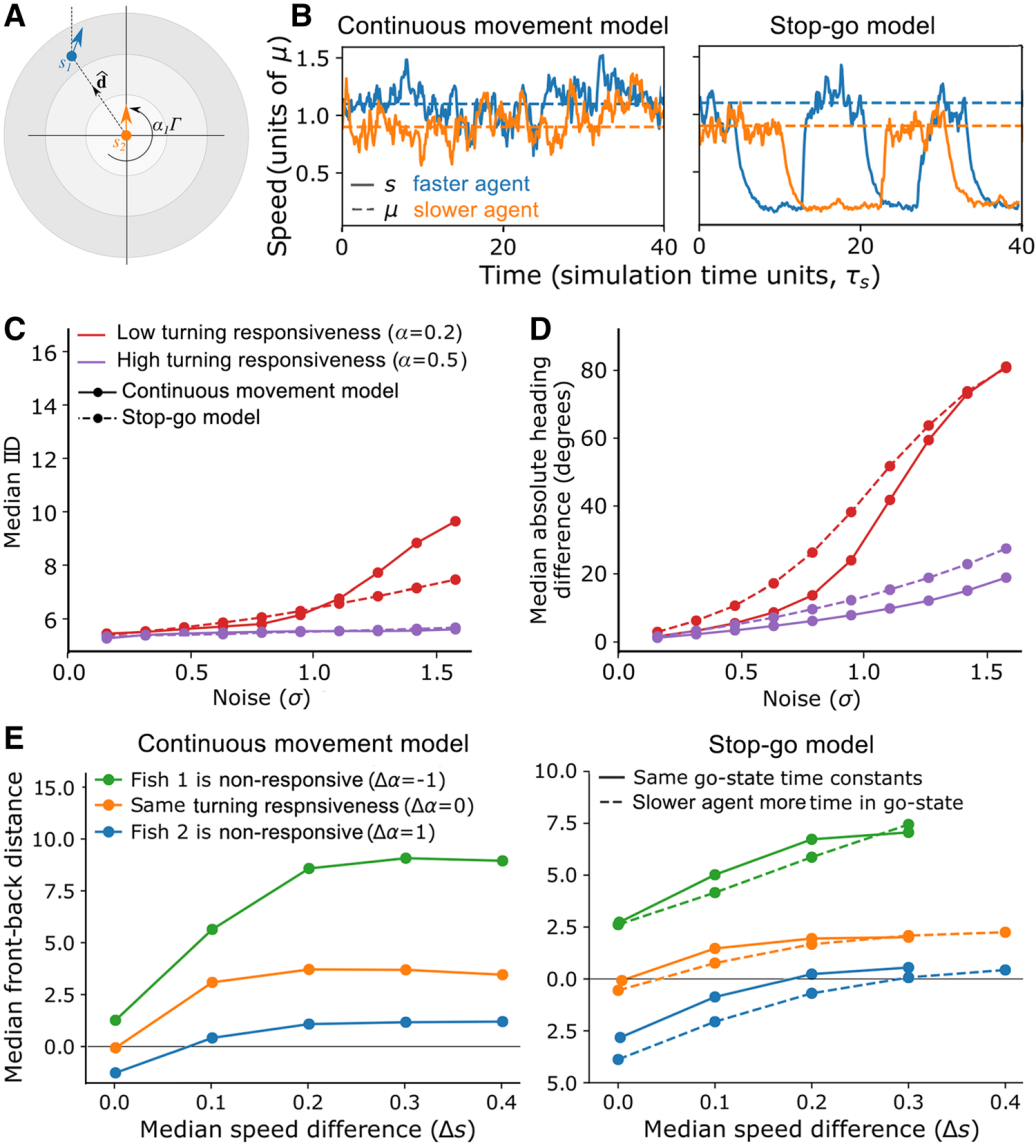
Agent-based simulations. (**A**) Illustration of the model showing two agents: the slower agent (orange) moving with speed ${s}_{2}(t)$, shown located at the origin, and the faster agent (blue 1) with speed ${s}_{1}(t)$. Agents experience a turning torque $\alpha \Gamma$ in the direction of $\widehat{d}$, which is the desired motion direction according to the zonal model (see Supplementary Material for details). (**B**) Two forms of our model, representing the case where agents are always moving (left) or when agents alternate between stop and go states (right). The parameter $\mu$ sets the average speed in the go state. (**C**,**D**) Group cohesion (**C**) and group alignment (**D**) as a function of turning noise shown for fish with low (red lines) and high (purple lines) turning responsiveness for both the continuous movement model (solid lines) and stop–go model (dashed lines). (**E**) Leadership results in terms of front/back distance when individuals differ in their speed and turning responsiveness for the continuous movement (left) and stop–go model (right) where agents have either the same (${T}_{go}=10$ for both; solid lines) or different values of the go state switching time constant (${T}_{go}=40$ for the slower agent, ${T}_{go}=10$ for the faster agent; dashed lines). Front/back distance is calculated with respect to the faster simulated fish (agent 1); a positive value indicates that fish 1 is the leader (i.e. spends more time in front). Similarly, the median speed difference is defined as $\Delta s={s}_{1}-{s}_{2}$, and the turning responsiveness difference is $\Delta \alpha ={\alpha }_{1}- {\alpha }_{2}$. Panels show leadership results as a function of median speed difference when agents have the same turning responsiveness of $\alpha =0.5$ (orange lines), compared to the extreme cases where agent 1 (faster agent) is non-responsive (${\alpha }_{1}=0$) and agent 2 (slower agent) is responsive (${\alpha }_{2}=1$) (green lines), or where the agent 2 is non-responsive and agent 2 is responsive (blue lines). See Supplementary Tables S1 and S2 for further model details.

To further gain mechanistic insights into how parasitism likely drives group-level effects, and thus to seek a parsimonious explanation for the observed patterns of cohesion, alignment, and leadership, we conducted individual-based simulations of self-organised groups (see Supplementary Material for model details). We varied individuals’ speed and turning behaviour (Fig. 5A), and considered cases where individuals either moved continuously or alternated between stop and go states (Fig. 5B), to reflect the differences we observed between infected and non-infected fish in our experiments. Simulations with pairs of agents showed that lower values of turning responsiveness resulted in a lower pair alignment (Fig. 5C) and, to a lesser extent, less cohesive pairs (Fig. 5D), as seen for the infected fish in our experiments. Spatial leadership was positively correlated with individual speed but negatively correlated with turning responsiveness and proportion of time in the stop state (Fig. 5E). This suggests that, based on the observed differences in speed, infected fish should be less likely to lead, as we observed, but contrastingly also that their slower turning speed and higher tendency to keep moving would make them more likely to lead. However, speed had the strongest effect and could thereby offset the opposite effects arising from the other two factors, explaining why infected fish spent tended to be less likely to be in front.

## Discussion

Using the stickleback-*S. solidus* system as a model, this study aimed to gain a mechanistic understanding of how parasite infection affects individual movement ability and thereby shapes group-level patterns. When tested individually, infected fish moved and accelerated more slowly than non-infected fish, both when free to move and when chased or startled. Infected fish also moved more continuously and were more predictable in their movements than were non-infected fish. We found that differences between infected and non-infected fish increased with increasing parasite load. In turn, parasite infection strongly affected fish’s social behaviour, with infected pairs moving more slowly and being less cohesive, less aligned, and less coordinated than pairs of non-infected fish, and mixed pairs being predominantly led by the non-infected individual. Using individual-based simulations of pairs of agents we reveal how these social patterns can emerge not from social interactions per-se, but rather due to individual differences in speed, turning ability, and stop–go behaviour linked to parasite infection.

The median movement speed of infected and non-infected fish was very similar across the solo trials, in line with previous work^14,28^. This may lead to the conclusion that parasite infection does not affect individual movement. However, detailed movement analyses revealed that speed distributions were bimodal, with two peaks corresponding to a moving and a non-moving state. Infected fish spent more time in the moving state but also moved at significantly lower cruising speeds compared to non-infected fish, and did not accelerate as strongly. These behavioural changes may benefit the parasite by increasing the chance its stickleback host will be eaten by its final host, a bird, and could therefore potentially signify host manipulation^41^. However, the parasite also imposes various energetic and physical constraints that could play a role. First of all, *S. solidus* increases its host’s metabolism^12–14^, and as a result, fish may voluntarily move at lower speeds to save energy. Secondly, the parasite grossly distends the body of their host, which impairs its streamlining and increases drag^11,13^. Indeed, previous work has shown that infected fish have higher increases in metabolism with increasing speed compared to non-infected fish^13,14^. This may explain why infected fish may not be able to sustain higher speeds. Third, *S. solidus* increases its host’s body volume and specific weight, as shown here (see Supplementary Material) and in previous work^10,12,13^. As the forward thrust required to change the inertia of a body in water is proportional to its volume and weight^42^, and is strongly affected by drag, fish heavily infected with *S. solidus* fish will be physically constrained in their acceleration, and increases in speed more energetically costly^43^. These mechanisms could potentially also explain why infected fish were more consistent in their speeds and less likely to stop moving completely (see also^23^), and increasingly so with higher parasite load.

When chased or startled, and thus pushed to their maximum, infected fish were much less able to accelerate and swim as fast as non-infected fish (see also^29,30^). This again suggests the parasite has a negative influence on the performance capacity of individuals by the physical constraints it imposes^32^. Indeed, experimental work with artificial parasites shows that it is especially the drag effects rather than metabolic effects that are important for reduced responses under such highly demanding conditions^44^. The finding that infected sticklebacks fled shorter distances after a simulated predator attack (see also^20^) therefore most certainly is a result of physical impairments, although also reduced risk avoidance behaviour by the parasite may play a role^19^. Besides affecting the speed, acceleration, and movement consistency of the fish, parasite infection was also found to affect turning behaviour, with infected fish exhibiting slower turning, here again, the effect being more pronounced for more heavily infected fish. This may be a further result of the distended abdomen of infected fish potentially increasing its body rigidity, which is known to be detrimental to manoeuvrability and turning performance^42,45^. This effect may potentially also help explain the observed finding that infected fish had weaker thigmotaxis, since increased rigidity would compromise fish’s ability to turn in response to a nearby wall, although this may also reflect a reduced anxiety of infected fish^21,40^.

The parasite-induced effects on individual movement were found in turn to influence social movement patterns. Besides pairs of infected fish tending to be slower than pairs of non-infected fish, they were also less cohesive, in line with previous work on fish infected with parasitic worms^7,33,34^, and less aligned, with mixed pairs showing intermediate levels of these behaviours. Such patterns could emerge naturally from the lower mobility of infected fish (i.e. lower speed/acceleration/manoeuvrability, see above) by the constraints it places on the individual to conform with its partner. This is supported by our analyses of the social response patterns, which showed that infected fish had weaker/slower responses to their partner than non-infected fish, and our simulations, which revealed that a lower turning responsiveness, as observed in infected fish, decreased group cohesion and alignment. Although the simulations do not differentiate between the physiological ability and social motivation to turn, the observation that infected fish were slower in turning when tested alone (in the solo assay) suggests that this difference in responsiveness is likely more biomechanical than a lower social motivation. This is in line with the finding that pairs of non-infected fish were not only more aligned than pairs of infected fish when moving, which could also arise directly from their higher speed^46,47^, but were also more aligned at very low speeds. Another potential explanation for the observed group-level patterns is that, because the parasite increases their host’s metabolism^14^, infected sticklebacks may have a higher motivation to search for food, since sticklebacks searching for food tend to be less cohesive^48^. However, in that case individual and group speeds of infected fish would be expected to be higher, and fish never experienced food in the testing environment. The abdominal swelling itself potentially directly impairs the ability of infected fish to accurately assess proximity (see also^34^), and explain why infected fish respond more slowly to, and stay further away from, others and the boundaries of their environment.

Parasite infection had a strong effect on the temporal coordination of the fish, with infected pairs being the least, and non-infected pairs the most, coordinated. The lower coordination of infected pairs may be a result of a reduced sociability of infected fish, which would again be beneficial to the parasite in making its host potentially more vulnerable to a predator. However, the results of the solo and pair context together also strongly suggest that infected fish have a physical impairment that negatively impacts their capacity to respond to others’ movements and thereby reduces coordination. Our finding that mixed pairs were primarily led by the non-infected fish can best be explained by self-organising effects from individual differences in speed as a result of parasite infection. Both our empirical data and our simulations showed a positive relationship between leadership and individual speed, which is conform previous work^46,49,50^. However, the simulations also revealed that leadership behaviour was positively linked to a lower turning responsiveness and a higher tendency to keep moving, aspects that we found to characterise infected fish, but which could be overruled by the effects of their much lower movement speed. The strong effects of parasite infection on group cohesion, alignment, coordination, and leadership may have important repercussions for the functioning of groups and the individuals within them, as they are linked to the vulnerability to predators, the ability to find food, and the rapid transfer of information^32,51^. Our results provide clear predictions for the effects of *S. solidus* on larger groups and among-group dynamics, with infected fish predicted to be less able to form stable, cohesive, coordinated groups, more likely to occupy positions on the edge and back of groups, and more likely to be isolated (see also^49^).

Many studies have previously shown that sticklebacks infected by *S. solidus* exhibited reduced predator avoidance behaviour and take more risks than do non-infected individuals^19,20,22,26,27^, which may enhance the transmission of the parasite to its final host. Here we provide quantitative results of two additional, rarely considered factors that may increase the predation risk of infected fish (see e.g.^52^). Specifically, we show that infected fish are less likely to stop moving completely and are slower to change in their speed and heading over time, as revealed by autocorrelation analyses, and are therefore more predictable in their movements. As a result, predators may both more easily detect infected compared to non-infected individuals, and better able to predict their future position. This may be especially important for many piscivorous birds, the final host of *S. solidus,* that hunt from above.

Together, our findings suggest that the changes in host morphology and physiology caused by the parasite lead to changes in individual and social behaviour that may in turn increase chances of transfer to its final host. Although our study did not explicitly test the potential for parasite manipulation, our results at least show that *S. solidus* might benefit from exploiting the cascading effects caused by its changes on host physiology and biomechanics. This is supported by our finding that the effects of the parasite increase with increasing parasite load (see also^31^^)^ rather than at a clear switch point in weight, which is linked to its potential to reproduce in its final host at which it starts to manipulate host behaviour^53^. However, it is clear that further empirical and theoretical work is needed that properly considers both active, i.e. the secretion of parasite chemical messengers that directly affect host brain function^54^, and passive, mechanisms to unravel the relative roles they may play in driving host behaviour.

A shortcoming of our study is that we worked with naturally infected sticklebacks, which makes it difficult to disentangle if some of the behavioural differences observed between infected and non-infected fish are not also correlated with the likelihood to be infected. For example, slower fish could potentially be more likely to eat infected copepods, and thus become infected, because of their slower speed. There is, however, some data that suggests at least body condition parameters do not differ between sham and parasite exposed but not infected fish^55^. It would furthermore be harder to explain why we find that behavioural effects increased with increasing parasite load, a factor that will be influenced by a number of factors, including time since infection and number of parasites in the body cavity. Still, it could be possible that some distinct host phenotypes are linked to both differences in behaviour and the suppression of parasite growth. However, such effects could also play a role in artificially infected sticklebacks when not carefully controlled for. It is clear future work is needed to fully elucidate to what extent fish’s behavioural phenotype and immune status interacts with their susceptibility to infection and its subsequent effects is clearly needed^9^.

Over a century ago, the complete complex life cycle of *S. solidus* was first discovered^56^. Since then we have gained incredible insights into this fascinating host-parasite model system. However, much about the effects of the parasite on the individual and social movements of its host had remained unclear. By systematically studying the movement dynamics of differently infected sticklebacks, and using a combination of empirical and theoretical approaches, we show that *S. solidus* has large and varied effects on the movements and social behavioural patterns of its intermediate host. These findings also contribute to the general understanding of the mechanisms by which parasites may affect host movement dynamics and the way in which individual differences in movement capacity can shape and impair group-level patterns. Exciting future work lies ahead to further investigate how parasitism may affect organismal movement at different social scales and thereby potentially shapes the structure and assortment of animal groups.

## Methods

### Experimental subjects

The three-spined stickleback (*Gasterosteus aculeatus*) is a small social species of fish, and a classic model system for the study of behavioural biology^57^, with individuals generally tending to shoal in small groups of tens of individuals but which occasionally range in the thousands. Fish were collected from one region in the littoral zone of Lake Konstanz during the summer using hand and dip nets. After catching, fish were immediately moved to our facilities and housed in large social housing tanks (100 cm length × 50 cm width × 50 cm height). Tanks were connected to a flow-through system with water coming directly from the lake from a depth of 12 m, resulting in a constant water temperature of ~ 13 °C. Standard illumination was provided from above and matched the day-night cycle. Fish were acclimated to the lab for at least three months before experiments. During this period, fish were fed defrosted bloodworms (Chironomidae) ad libitum once daily.

### Experiment 1

We randomly selected 180 fish from the social housing tanks, visually controlling for size (mean ± SE = 40.00 ± 0.32 mm), and moved them to our experimental lab. To be able to create specific pair combinations of infected and non-infected fish, individuals were anaesthetised using MS222, photographed from above using a tripod-mounted camera, and their infection status predicted based on their abdomen shape and size^16,17^. As no clear phenotypic differences exist between male and female sticklebacks under the laboratory conditions, the sex of the fish was not determined. To enable individual identification, fish were given a small coloured plastic tag with written id number on their first dorsal spine^58^, after which they were left to recover in buckets of fresh, aerated water.

Fish were housed in five identical glass aquaria, each consisting of three compartments of equal size (20 × 40 × 40 cm; 34 cm depth), separated by mesh partitions, and with a fresh influx of lake water at 13 ± 1 °C. Fish were randomly allocated to compartments in groups of 16 fish, and identifiable by their tag colour-id combination. After the experiments, all fish were euthanised with an overdose of MS222 (1 g/l), weighed, their body cavity opened, and plerocercoids removed and weighed individually to the nearest mg. Based on the weight of the fish and their parasites we then determined the parasite load for each fish: the proportion of body weight attributable to parasites^18,20,29^ (for further descriptives about the fish and parasites, see Supplementary Fig. S1). Comparing the infection status of the fish as determined pre- and post-mortem revealed a prediction accuracy of 97%, with all infected fish predicted correctly.

The experimental period started after fish were housed in the experimental holding tanks for five days, during which fish were fed defrosted bloodworms ad libitum to allow for acclimation and stabilization of any social modulation effects^59^. Experiments were conducted using three identical white Perspex open-field arenas (50 × 70 × 10 cm; water depth: 8 cm). Arenas were placed inside a large, covered structure to minimise any external disturbances and to provide diffuse lighting. Trials were recorded from above with Raspberry Pi computers (RS Components Ltd) using the pirecorder package^60^. For experiment 1, we started by testing fish twice individually in the open arena (‘solo assay’; days 1 and 5). Fish were netted from their holding compartment and moved to the experimental arena where they were left to acclimate in a transparent cylinder (10 cm diameter) in the centre of the tank. After one minute, the trial started and the cylinder was remotely lifted by the experimenter, and fish were allowed to explore the arena for 10 min. After the trial, fish were fed individually by moving them to a glass container containing two bloodworms before being returned to their housing compartment. The order in which fish were tested was randomised but the same order was used for both testing days of the solo assay.

After three more rest days (day 9), fish were retested again but now in pairs (‘pair assay’). Individuals were randomly allocated to pairs based on their infection status to get roughly equal numbers of pairs in which no fish was infected (pair status: NN), only one fish was infected (pair status: NI), and both fish were infected (pair status: II). Due to some false negatives, determined from the post-mortem analyses (see above), and the loss of one fish, the actual pair numbers differed slightly between the treatment groups (*n* = 17, 21, and 21 pairs respectively). The proportion of individuals with low and high parasite load was roughly the same between the NI and II pairs (45.1% and 45.9% respectively). As differences in body size have previously been shown to influence social interactions in fish^61^, we further assorted fish based on their standard length (henceforth ‘body length’; BL), resulting in a negligible length difference between paired fish (1.66 ± 0.17 mm; see Supplementary Fig. S2). Trials again lasted for 10 min.

### Experiment 2

To investigate how parasite infection alters the maximum movement capacity of individual fish, we conducted an additional experiment and tested naïve fish under the demanding conditions of being chased and startled. 24 size-matched individuals (32.8 ± 0.4 mm), 11 of which were infected (confirmed post-mortem) were taken from the social housing tanks and moved to individual holding compartments (20 × 10 cm, enabling visual and chemical social cues) in our experimental lab. After two days of acclimation, each fish was subjected to a ‘startle assay’ and a ‘chase assay’ twice on two subsequent days (see Supplementary Figs. S3 and S4). For the chase assay, fish were acclimated to the experimental tank for one minute after which they were briefly chased with a net that was moved at fish’s maximum speed before being caught. For the startle assay we simulated a predator attack with a fake heron-like structure 50 cm above the water following previous work^20,21,27^. Fish were released into the tank and after at least 30 s, the structure was remotely triggered the moment a fish entered that side of the tank, resulting in its immediate release into the water. Our aim with this assay was to determine how fish differed in their maximum movement capacity, not their escape potential of a predator, for which other standardized assays exist (see^45^). Chase and startle tests were conducted under similar conditions as Experiment 1, i.e. an open, familiar Perspex tank (50 × 100 cm) in a covered structure, but trials were recorded with a GoPro camera fixed above the tank.

### Data processing

We used custom-developed tracking software to acquire detailed coordinate data (centre of mass) and orientation (angle of the head) of the fish. For the solo and pair assays, fish were automatically tracked while for the chase and startle assays this was done by manual tracking, resulting in four chase trajectories and two startle trajectories for each fish. Coordinates were converted from pixels to mm and subsequently smoothed using a Savitzky-Sgolay smoothing filter. After tracking, all trajectories were visually checked for any inconsistencies or errors.

From the tracking data, we computed each fish’s velocity, speed, acceleration, and turning speed. We also looked at ‘thigmotaxis’, the proportion of time fish spent within 5 cm of the nearest wall, which may indicate risk-avoidance behaviour^40^ but also potentially result from a lower ability to turn. Measures linked to movement speed were computed in terms of body lengths to account for any effects of size. We estimated individuals’ movement tendency by computing the proportion of time spent moving, using a criterion of > 0.5 BL/s to determine if a fish was moving or not, based on the speed distribution (see Fig. 2A). To estimate fish’s cruising speed, we quantified their median speed when in this ‘moving state’. Neither the tendency to move or fish’s movement speed were correlated with their distance from the tank walls (Supplementary Fig. S5). As a measure of turning ability we computed the average time it took a fish to turn its body at least 15°. We also estimated the predictability of fish’s movements, which may decrease an animal’s ability to avoid predation, by computing temporal autocorrelations of fish’s speed and heading. For the chase and startle assay, we also computed the 0.75th quantile of fish’s speed and acceleration as a measure of their ‘maximum’ speed/acceleration. This was done to overcome potentially characterising fish based on single frames with relatively extremely high speed/acceleration.

For the pair trials we additionally computed the position and movement vector (between frames) of the group centroid (based on its centre of mass). We then calculated each pairs’ cohesion, in terms of the distance between the two fish, alignment, in terms of the absolute difference in orientation, and group speed, in terms of the speed of the centroid. We also computed fish’s front-back positioning relative to the group centroid when fish were close together, to determine which individual in the mixed pairs was more likely to lead. Based on visual inspections of the data we used a criterion of 1.5 BL side-by-side and 2.25 BL front-to-back (see Fig. 4F). Finally, to determine the propagation of movement changes in the pairs, we ran temporal correlations on their speed and heading, and determined the average directional correlation between the two fish as a function of the delay in time^46,62^. A detailed explanation of the computation of these behavioural measures can be found in the Supplementary Material. For all variables we computed either the mean or median, based on the distribution of the raw data, and inter-quartile range on an individual- and pair-basis.

### Statistical analysis

To investigate the role of parasite infection on individual and pair movement dynamics we used a generalised linear mixed modelling (LMM) approach. The models of experiment 1 included as fixed effects trial (single assay only), parasite load, categorised into a no (*n* = 84), low (0.002–0.27; *n* = 47), and high (0.27–0.49; *n* = 47) category (see Supplementary Fig. S1 for further details), and total body mass. We used parasite load as a categorized variable to be able to investigate the role of fish’s infection status and parasite load in the same model. Fish id was added as a random factor, which was further nested within group id for the paired trials. For the chase and startle assays we ran separate models with 0.75th speed quantile and 0.75th acceleration quantile as response variables, infection status (non-infected, infected) and test (chase, startle) as fixed factors, and fish id nested within trajectory as a random factor. Infection status rather than parasite load category was used to account for the smaller sample size of experiment 2. To investigate leadership in the mixed pairs, we ran a one-sided t-test to determine if infected fish on average spent more time in front and a chi-square test to determine if the majority of pairs led by non-infected fish was higher than chance level. For our LMM approach we generally used models fitted with a Gaussian error distribution except for count data, which was fitted to a Poisson error distribution with log-link function. Minimal adequate models were obtained using backward stepwise elimination, i.e., sequentially dropping the least significant terms from the full model, until all terms in the model were significant. Statistics for non-significant terms were obtained by adding the term to the minimal model. Neither housing compartment nor tank number had an effect on any of the behaviours analysed. Residuals were visually inspected to ensure homogeneity of variance, normality of error and linearity where appropriate; data was square-root or log-transformed otherwise. Means are quoted ± SE unless stated otherwise. All data were analysed in R 3.5.0. A mathematical description and details of the individual-based model simulations can be found in the Supplementary Material.

### Ethical statement

The experimental procedures were approved by Regierungspräsidium Freiburg (G-16/144, G-19/50) and were carried out following the guidelines for the Use of Animals in Research of the Association for the Study of Animal Behaviour (ASAB/ABS).

## Supplementary information

Supplementary Information 1.

## Data Availability

Data accompanying this paper can be found in the Mendeley Data repository (10.17632/gjr8gxk6nw.1). Python code for running the individual-based simulations can be found on GitHub (https://git.io/Je6OT).
